# Therapeutic Potential of Autologous Adipose-Derived Stem Cells for the Treatment of Liver Disease

**DOI:** 10.3390/ijms19124064

**Published:** 2018-12-15

**Authors:** Chiara Gardin, Letizia Ferroni, Gloria Bellin, Giuseppe Rubini, Simone Barosio, Barbara Zavan

**Affiliations:** 1 Department of Biomedical Sciences, University of Padova, Via Ugo Bassi 58/B, 35131 Padova, Italy; chiara.gardin@unipd.it (C.G.); gloria.bellin@gmail.com (G.B.); 2 Maria Cecilia Hospital, GVM Care & Research, E.S: Health Science Foundation, Via Madonna di Genova 1, 48033 Cotignola, Italy; 3 Ultravet Diagnostic, Via Enrico Fermi 59, San Giovanni in Persiceto, 40017 Bologna, Italy; giusepperubini@ymail.com (G.R.); simocla16@yahoo.it (S.B.)

**Keywords:** adipose-derived stem cells (ADSCs), liver disease, immunomodulatory properties, autologous cell transplantation

## Abstract

Currently, the most effective therapy for liver diseases is liver transplantation, but its use is limited by organ donor shortage, economic reasons, and the requirement for lifelong immunosuppression. Mesenchymal stem cell (MSC) transplantation represents a promising alternative for treating liver pathologies in both human and veterinary medicine. Interestingly, these pathologies appear with a common clinical and pathological profile in the human and canine species; as a consequence, dogs may be a spontaneous model for clinical investigations in humans. The aim of this work was to characterize canine adipose-derived MSCs (cADSCs) and compare them to their human counterpart (hADSCs) in order to support the application of the canine model in cell-based therapy of liver diseases. Both cADSCs and hADSCs were successfully isolated from adipose tissue samples. The two cell populations shared a common fibroblast-like morphology, expression of stemness surface markers, and proliferation rate. When examining multilineage differentiation abilities, cADSCs showed lower adipogenic potential and higher osteogenic differentiation than human cells. Both cell populations retained high viability when kept in PBS at controlled temperature and up to 72 h, indicating the possibility of short-term storage and transportation. In addition, we evaluated the efficacy of autologous ADSCs transplantation in dogs with liver diseases. All animals exhibited significantly improved liver function, as evidenced by lower liver biomarkers levels measured after cells transplantation and evaluation of cytological specimens. These beneficial effects seem to be related to the immunomodulatory properties of stem cells. We therefore believe that such an approach could be a starting point for translating the results to the human clinical practice in future.

## 1. Introduction

Liver diseases are a widespread problem for national health care systems over the world. In case of hepatic failure, the only effective approach is organ transplantation, but its practical application is constrained by the limited availability of donor organs, immunological side effects, and economic reasons [1]. The development of alternative methods for treatment of liver disease is highly requested. In this context, the emerging field of regenerative medicine offers new approaches based on cell transplantation or organ engineering. Although organ engineering has the potential to solve the problem of liver donor shortage, cell transplantation is a much less invasive and expensive approach. This consists in the transfusion or injection of cells aimed at increasing the regeneration capacity in recipient liver and improving the restoration of its structure and function [2]. Isolated hepatocytes have been used as the primary cell source for cell transplantation procedures; nevertheless, hepatocytes do not survive for prolonged period in in vitro culture, and after cryopreservation their functions are dramatically impaired [3]. However, the main limitation remains the scarcity of donor livers from which high-quality primary hepatocytes can be isolated [4].

Mesenchymal stem cells (MSCs) have recently gained attention as potential candidates for liver regeneration [5]. MSCs transplanted in diseased liver can transdifferentiate into liver cells after homing within liver tissue, and/or stimulate tissue regeneration by paracrine secretion of cytokines, chemokines, and growth factors [6]. Notably, MSCs possess other useful properties, including low immunogenicity, affinity to the injury sites, and ability to modulate immune responses by downregulating T cells, B cells, and natural killer cells function [7]. Several reports have shown that the application of autologous or allogeneic MSCs are useful for improving acute and chronic liver failure including liver fibrosis in animal models [8,9,10]. Consequently, most of preclinical and clinical research in the field of cell therapy in the liver is currently conducted with MSCs, in particular with those derived from adipose tissue.

The human liver has a greater similarity with the canine liver rather than with rodent liver [11]. Normal canine and human livers show the same morphology, histological organization, and identical expression pattern of markers for mature hepatocytes and progenitor cells. Moreover, liver diseases in dogs occur spontaneously as in humans, and in both species acute and chronic hepatitis display an equal morphological reaction pattern [12]. Unlike humans, chronic hepatitis in dogs represents a pathological condition with almost non-specific symptoms and the diagnosis can only be confirmed on a histopathological basis, but fibrosis is considered the common evolution in the two species. By considering liver reaction similarities in the two species, comparative studies could yield useful data to increase the knowledge on liver diseases bilaterally.

In this perspective, in the study presented here we analyzed and compared specific features of MSCs isolated from canine and human adipose tissue. As far as we know, this is the first study in which several morphological and functional characteristics of both canine and human MSCs are compared in the same work. Firstly, we evaluated the stemness profile of both cell populations investigating phenotype morphology, expression and frequency of surface markers. Then, we examined their growth rate and proliferation under standard culture conditions or after simulating transportation conditions. The in vitro differentiation potential of both cell populations was further established by quantification of gene expression and histological staining. Finally, dogs with spontaneous chronic liver disease were treated with autologous undifferentiated MSCs in order to evaluate their ability to restore liver function.

## 2. Results

### 2.1. Isolation and Characterization of Canine and Human Stem Cells

**Figure 1:**
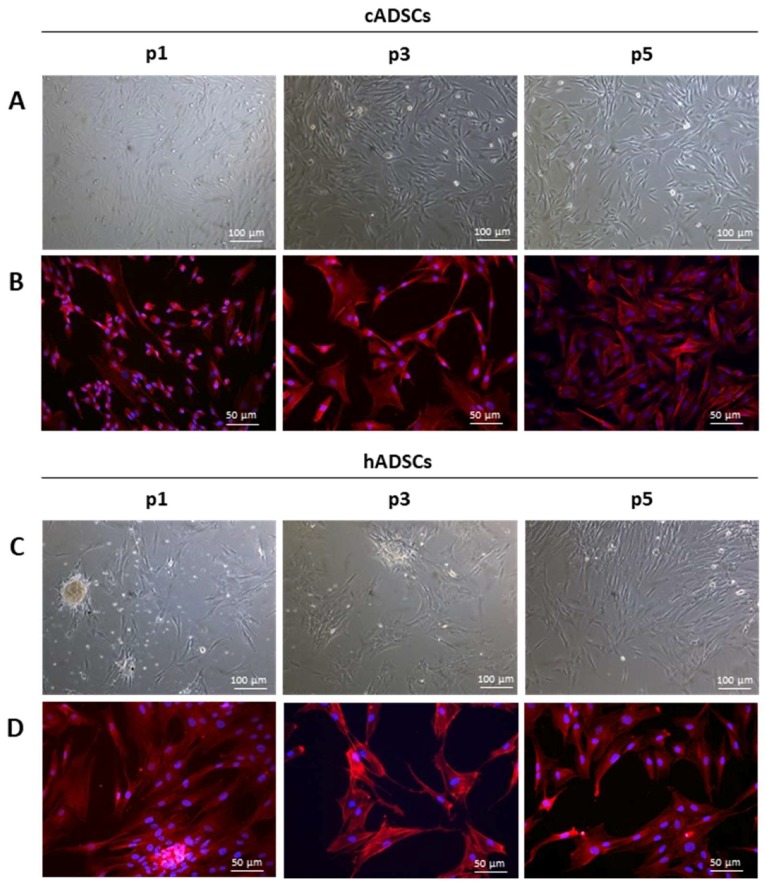
Morphology of canine adipose-derived stem cells (cADSCs) and human ADSCs (hADSCs). (**A**,**C**) Phase-contrast microscopy images (magnification 10×) showing the morphology of cADSCs and hADSCs, respectively, at passage 1 (p1), p3, and p5. (**B**,**D**) Actin cytoskeleton staining (magnification 20×) with phalloidin (in red) of cADSCs and hADSCs, respectively, at p1, p3, and p5. Nuclei are counterstained with Hoechst (in blue).

Freshly isolated canine and human MSCs derived from fat biopsies were plated under basal conditions in tissue culture flasks. Both cell populations were adherent to the plastic, and displayed a spindle-shaped fibroblast-like morphology, which was maintained with cell passaging (Figure 1A,C). At early passages, human adipose-derived stem cells (hADSCs) formed several colonies, which disappeared with subsequent passages; these colonies were not present in the canine cell population at passage 1 (p1). Immunostaining of the actin cytoskeleton with phalloidin better revealed the morphology of canine ADSCs (cADSCs) and hADSCs (Figure 1B,D). Compared to hADSCs, primary canine cell cultures included smaller cells as well as spindle-shaped fibroblastic cells. This heterogeneity decreased with subsequent passages as the spindle-shaped fibroblastic cells appeared to predominate.

**Figure 2:**
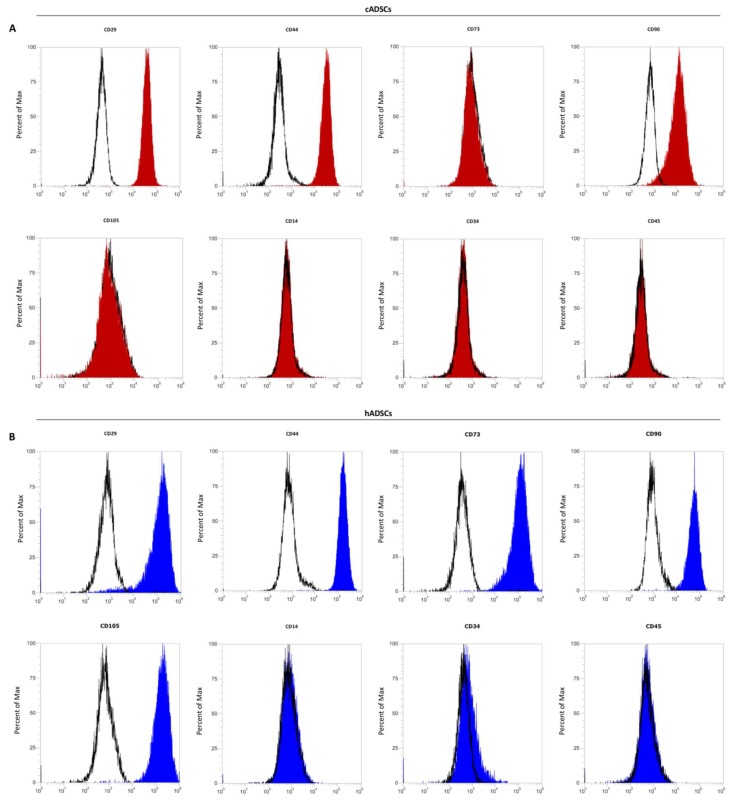
Characterization of cell surface markers in cADSCs and hADSCs by flow cytometry. (**A**) cADSCs are positive to CD29, CD44, and CD90, and negative to CD73 and CD90. (**B**) hADSCs show positivity to CD29, CD44, CD73, CD90, and CD105. Both cell populations are negative to CD14, CD34, and CD45.

Cell surface antigens characterization was then performed on cADSCs and hADSCs by flow cytometry. The two cell populations displayed very similar expression patterns of surface markers. In particular, cADSCs were positive to the MSCs markers CD29, CD44, and CD90, and negative for the hematopoietic markers CD14, CD34, and CD45 (Figure 2A). Negative expression of CD73 and CD105 was also found in the canine cell population. hADSCs showed positivity to CD29, CD44, CD73, CD90, and CD105, and negativity to CD14, CD34, and CD45 (Figure 2B). Percentages of MSCs surface marker expression for both canine and human cells are reported in Table 1.

### 2.2. Proliferation, Growth Rate, and Colony Formation of Canine and Human Stem Cells

**Figure 3:**
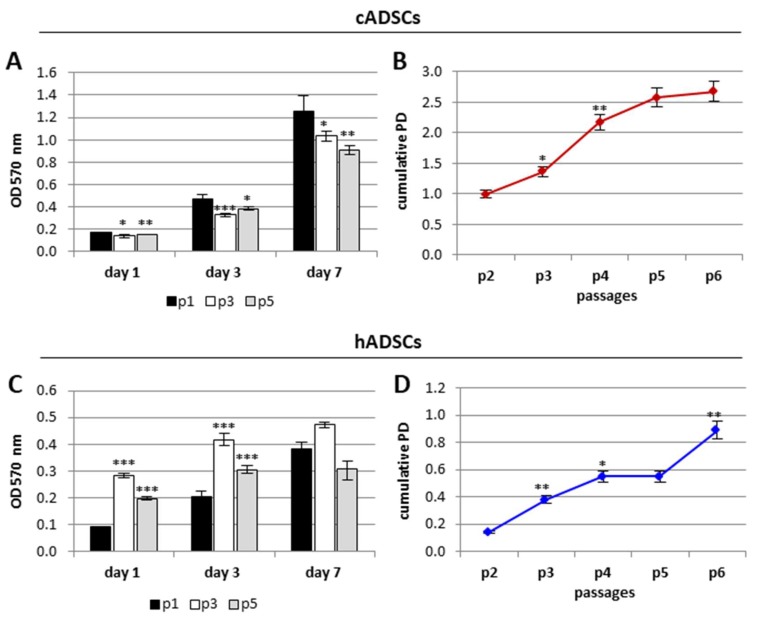
Proliferation rate of cADSCs and hADSCs. (**A**,**C**) MTT assay of cADSCs and hADSCs, respectively, at p1, p3, and p5 at days 1, 3, and 7 post seeding. Data are expressed as mean ± SD (*n* = 3). * *p* < 0.05, ** *p* < 0.01, *** *p* < 0.001 indicate statistically significant difference compared to cells at p1. (**B**,**D**) Cumulative Population Doubling (PD) of cADSCs and hADSCs, respectively, from p2 to p6. PD is measured at each passage. Data are expressed as mean ± SD (*n* = 3). * *p* < 0.05, ** *p* < 0.01, *** *p* < 0.001 indicate statistically significant difference compared to cells at the previous passage.

The proliferation rate of the two cell populations was evaluated by means of the Methyl Thiazolyl-Tetrazolium (MTT) assay. As shown in Figure 3A, cADSCs proliferation increased over seven days of culture; nevertheless, when comparing cell passages, proliferation rate was significantly higher for cells at p1 compared to p3 and p5. Regarding human cells, an increase in cell proliferation during culture time was observed for cells at p1 and p3; on the contrary, hADSCs at the highest passage showed a slowdown in proliferation after seen days from seeding (Figure 3C).

A population doubling (PD) assay was additionally performed to establish growth potential of canine and human cells during six consecutive passaging. The cumulative PD, which corresponds to the total number of estimated divisions up to that passage, tended to be higher for cADSCs respect to hADSCs at all passages examined (Figure 3B). Compared to cADSCs, hADSCs were indeed characterized by a lower rate of cell doublings (Figure 3D).

**Figure 4:**
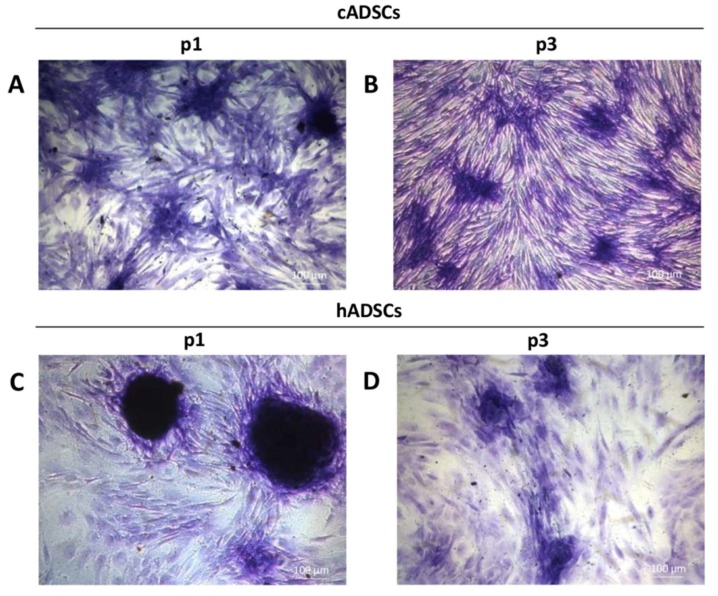
Representative images of Colony Forming Units-Fibroblast (CFUs-F) morphology of cADSCs and hADSCs after eight days of culture. (**A**,**B**) Toluidine blue staining (magnification 10×) of colonies generated by cADSCs at p1 and p3, respectively. (**C**,**D**) Toluidine blue staining (magnification 10×) of colonies generated by hADSCs at p1 and p3, respectively.

In order to determine the ability of the canine and human cell populations to form clonal fibroblastic colonies, a limiting dilution colony forming units-fibroblast (CFUs-F) assay was performed. As expected, both cADSCs and hADSCs formed more fibroblastic colonies as seeding densities increased. There were no significant differences in the CFUs-F frequencies between cell populations at the same passage. In detail, the frequency of precursor cells was 1/(1.92 × 10^3^ ± 27) for cADSCs at p1, and 1/(1.86 × 10^3^ ± 32) for hADSCs at the same passage. (Table 2). For both canine and human cells, p3 CFUs-F frequencies were lower than for p1 cells. As shown in Table 2, MSCs frequencies at p3 were 1/(2.34 × 10^3^ ± 26) for cADSCs and 1/(2.18 × 10^3^ ± 28) for hADSCs. Regarding the morphology of the colonies, those generated from hADSCs (Figure 4C,D) were more dense and larger in size compared to the canine colonies (Figure 4A,B).

### 2.3. In Vitro Osteogenic and Adipogenic Differentiation of Canine and Human Stem Cells

**Figure 5:**
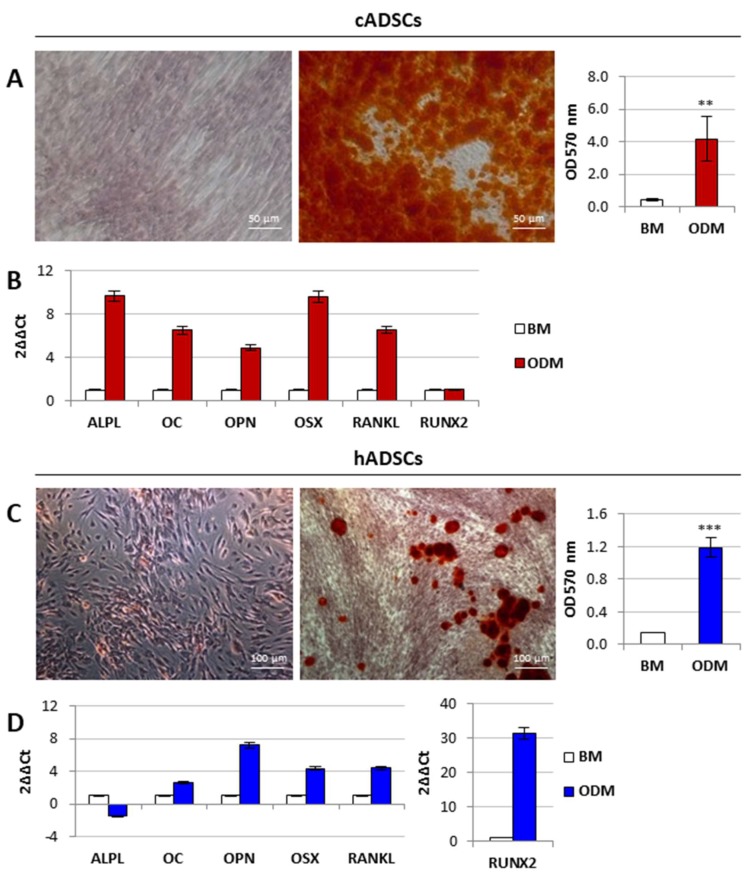
In vitro osteogenic differentiation potential of cADSCs and hADSCs. (**A**,**C**) Alizarin Red S (ARS) staining and quantification of calcium deposits in cADSCs (magnification 20×) and hADSCs (magnification 10×), respectively, after 21 days of osteogenic differentiation in osteogenic differentiation medium (ODM). Data are expressed as mean ± SD (*n* = 3). ** *p* < 0.01, *** *p* < 0.001 indicate statistically significant difference compared to cells grown in Basal Medium (BM). (**B**,**D**) Gene expression profiles of the osteogenic markers ALPL, OC, OPN, OSX, RANKL, and RUNX2 in cADSCs and hADSCs, respectively.

cADSCs and hADSCs at p3 were induced to differentiate into osteogenic and adipogenic lineages by appropriate culture media. Osteogenesis was assessed with visual staining of calcium deposits followed by quantification of extracted Alizarin Red S (ARS), and evaluating the expression profile of selected osteogenic markers (Figure 5A–D). After 21 days of osteogenic differentiation, canine cells took a more round polygonal appearance than those cultured in basal medium (BM), and large mineralized nodules appeared (Figure 5A). ARS accumulation in cells grown in osteogenic differentiation medium (ODM) was robust when compared to the undifferentiated condition, and a significant (*p* < 0.01) increase in ARS extraction was detected (Figure 5A). cADSCs maintained in ODM for 21 days expressed higher mRNA levels of alkaline phosphatase (*ALPL*), osteocalcin (OC), osteopontin (OPN), osterix (OSX), and TNF superfamily member 11 (RANKL) than cells maintained in BM for the same culture time. No difference for runt related transcription factor 2 (RUNX2) expression was observed (Figure 5B). Also, the human stem cells population displayed a round polygonal shape but smaller mineralized nodules were visible after 21 days in ODM. Nevertheless, ARS accumulation in hADSCs cultured in ODM was higher than the control condition (hADSCs in BM), and a significant (*p* < 0.001) increase in ARS extraction was measured (Figure 5C). OC, OPN, OSX, RANKL, and RUNX2 mRNAs were more expressed in hADSCs grown in ODM than in uncommitted cells. On the contrary, ALPL expression was lower in hADSCs in ODM than in BM (Figure 5D).

**Figure 6:**
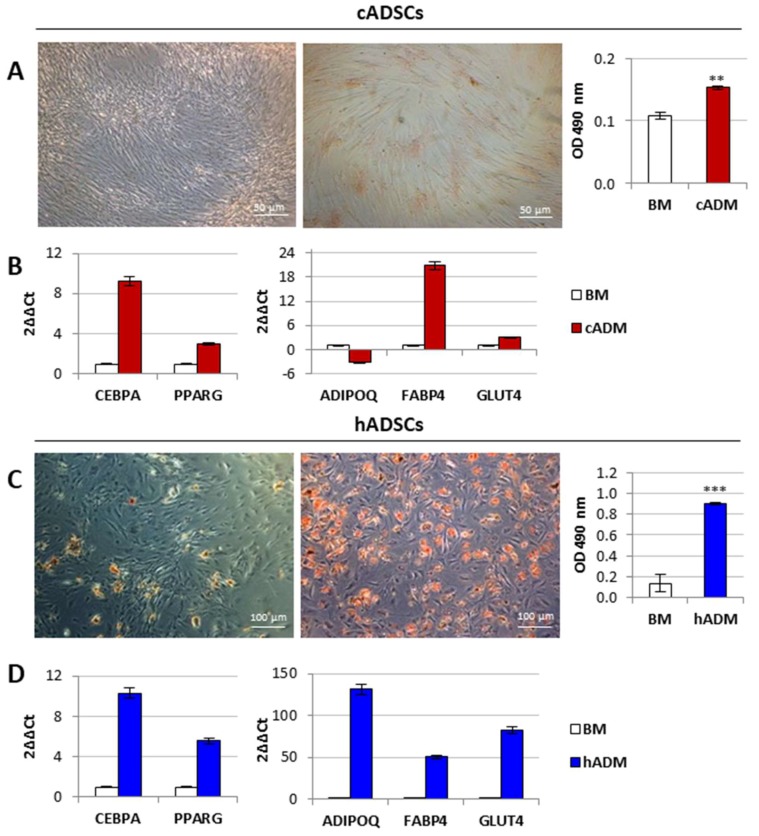
In vitro adipogenic differentiation potential of cADSCs and hADSCs. (**A**,**C**) Oil Red O (ORO) staining and quantification of lipid droplets in cADSCs (magnification 20×) and hADSCs (magnification 10×) after 21 and 14 days of adipogenic differentiation, respectively, in adipogenic differentiation medium (ADM). Data are expressed as mean ± SD (*n* = 3). ** *p* < 0.01, *** *p* < 0.001 indicate statistically significant difference compared to cells grown in BM. (**B**,**D**) Gene expression profiles of the adipogenic markers ADIPOQ, CEBPA, FABP4, GLUT4, and PPARG in cADSCs and hADSCs, respectively.

Adipogenesis was evaluated by both visual assessment of lipid vacuole accumulation and quantification of Oil Red O (ORO) staining, and gene expression profile of adipogenic markers (Figure 6A–D). Considering cADSCs, adipogenic differentiation was observable in a very limited number of cells; nevertheless, a significant (*p* < 0.01) increase in ORO extraction was detected with respect to the undifferentiated cells (Figure 6A). The mRNA levels of CCAAT enhancer binding protein alpha (CEBPA), fatty acid binding protein 4 (FABP4), solute carrier family 2 (facilitated glucose transporter) member 4 (GLUT4), and peroxisome proliferator activated receptor gamma (PPARG) were higher in cADSCs grown in canine adipogenic differentiation medium (cADM) for 21 days compared to cells maintained in BM. The expression of adiponectin (ADIPOQ) was instead lower in cADM when compared to the undifferentiated control condition (Figure 6B). Lipid vacuole staining was observed in all hADSCs after 14 days in human ADM (hADM), and ORO extraction was significantly (*p* < 0.001) greater in this condition than in uncommitted cells (Figure 6C). Gene expression profiles of all the adipogenic markers listed above resulted higher in cells cultured in hADM than in the control condition (Figure 6D).

### 2.4. Preservation of Canine and Human Cells under Simulated Transportation Conditions

**Figure 7:**
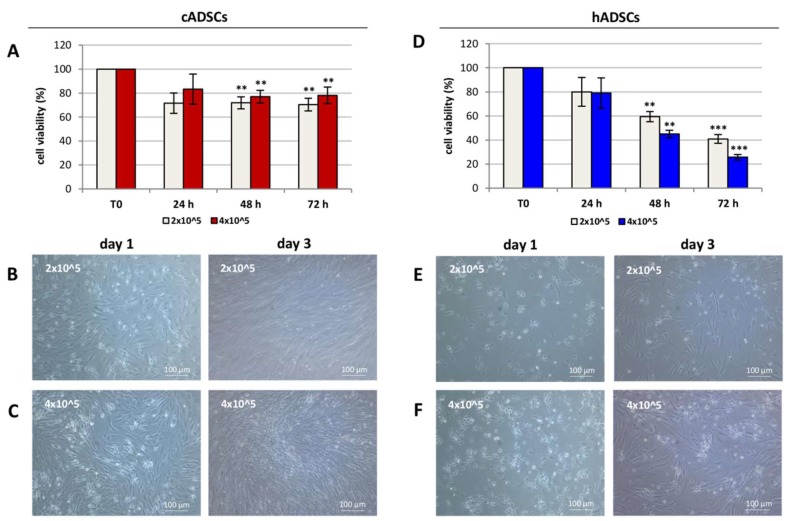
Percentage viability of cADSCs and hADSCs under simulated transportation. (**A,D**) Percentage viability of cADSCs and hADSCs, respectively, after 24, 48, and 72 h of simulated transportation. Data are expressed as the mean ± SD (*n* = 3). * *p* < 0.01, *** *p* < 0.001 indicate statistically significant difference compared to cells at T0. (**B**,**C**) Phase-contrast microscopy images (magnification 10×) showing proliferation of (2 × 10^5^ and 4 × 10^5^) cADSCs at day 1 and day 3 after recovering. (**E**,**F**) Phase-contrast microscopy images (magnification 10×) showing proliferation of (2 × 10^5^ and 4 × 10^5^) hADSCs at day 1 and day 3 after reseeding.

High viability (between 70–80% of the initial cell number) was observed in cADSCs after 24 h of simulated transportation conditions (Figure 7A). This degree of viability was preserved during the following two days for both tested doses. Considering the human stem cell population, high viability (around 80%) was measured at 24 h; nevertheless, a significant reduction of cell viability (between 25–60%) was registered at 48 h (*p* < 0.01) and 72 h (*p* < 0.001) from the mimicked transportation conditions (Figure 7D). This scenario was reflected in phase-contrast images, where canine cells (Figure 7B,C) showed a greater confluence than human cells (Figure 7E,F) for both tested doses at day 1 and 3 after recovering.

### 2.5. Transplantation of Autologous cADSCs in Dogs and Evaluation of Liver Biomarker Levels

**Figure 8:**
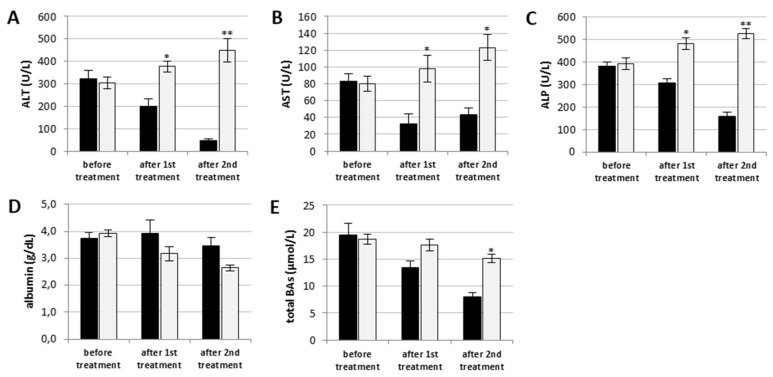
Serum levels of liver biomarkers. Serum levels of (**A**) ALT, (**B**) AST, (**C**) ALP, (**D**) albumin, and (**E**) total BAs were measured in blood samples collected before injection and seven days after each transplantation. Black bars represent the transplanted group; grey bars relate to the PBS-treated control group. In healthy dogs, the levels of ALT, AST, ALP, and total BAs should be less than 120 U/L, 50 U/L, 130 U/L, and 12.5 µmol/L, respectively. For albumin, the normal range is between 2 and 4 g/dL. Data are expressed as mean ± standard deviation. * *p* < 0.05, ** *p* < 0.01 indicate statistically significant difference compared to levels recorded in the control group.

Ten dogs suffering from severe liver disease were subjected to adipose tissue aseptical collection at suprascapular or interscapular region. From each fat sample, cADSCs were isolated and amplified in 21 days. Then, autologous cells were transfused through portal vein under ultrasound guidance. Each dog underwent two inoculations 30 days apart. To evaluate the effects of cADSCs transplantation on clinical outcomes and biochemical functions, levels of serum alanine aminotransferase (ALT/GPT), aspartate aminotransferase (AST/GOT), alkaline phosphatase (ALP), albumin, and total bile acids (BAs) were measured. Compared to the values recorded before treatments, a reduction in ALT, AST, ALP, and total BAs levels was detected after the first and second injection; levels of the same markers were also significant lower than those of the phosphate-buffered saline (PBS)-treated control group (Figure 8A,B,C,E). No significant alteration in serum albumin was observed (Figure 8D).

### 2.6. Immunomodulatory Properties of Canine and Human Stem Cells

**Figure 9:**
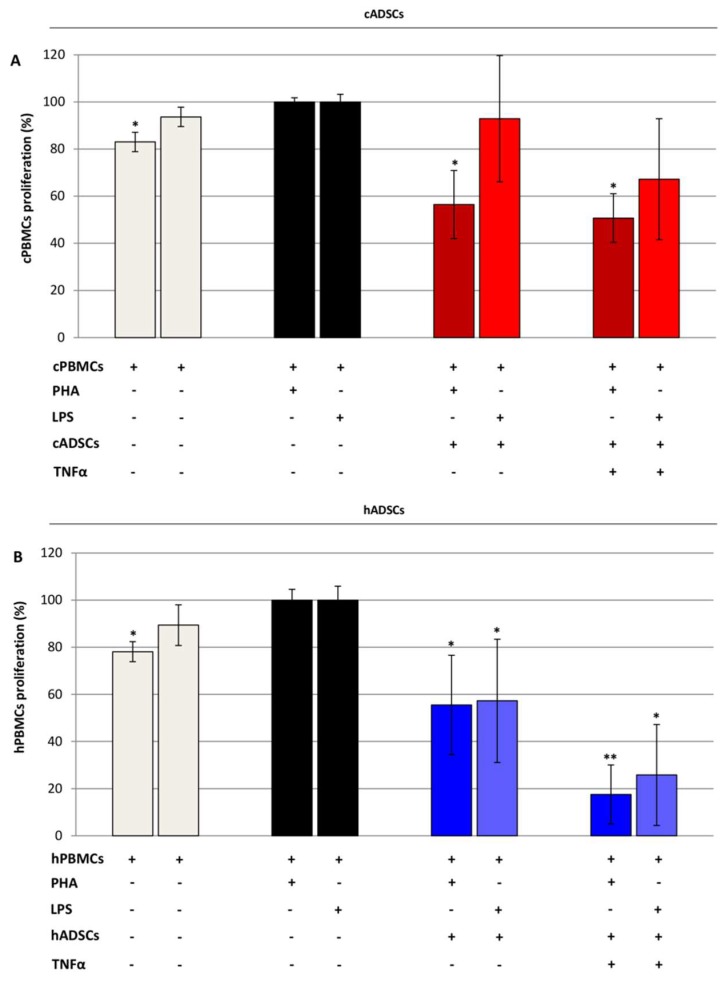
Proliferation of canine and human peripheral blood mononuclear cells (PBMCs) in co-culture with allogenic ADSCs (10:1 ratio). (**A**) MTS assay of cPBMCs after three days of co-culture with allogenic cADSCs at p3. cPBMCs were stimulated with phytohemagglutinin (PHA) or lipopolysacchride (LPS). (**B**) MTS assay of hPBMCs after three days of co-culture with allogenic hADSCs at p3. hPBMCs were PHA- or LPS-stimulated. The graphs report the proliferation percentage compared to stimulated PBMCs (100% proliferation). Data are expressed as mean percentage ± SD (*n* = 3). * *p* < 0.05, ** *p* < 0.01 indicate statistically significant difference with respect to stimulated PBMCs.

In order to examine whether canine stem cells could inhibit the proliferation of peripheral blood mononuclear cells (PBMCs) following mitogen stimulation, cADSCs pre-treated with mitomycin C were co-cultured with phytohemagglutinin (PHA)- or lipopolysacchride (LPS)-stimulated allogenic canine PBMCs (cPBMCs) at a 1:10 ratio for three days. For the sake of comparison, the same procedure was applied to the human cells’ counterpart. As shown in Figure 9A, cADSCs were able to inhibit cPBMC proliferation in response to PHA; similar results were obtained for the human cells (Figure 9B). Notably, we observed a significant (*p* < 0.05) decrease in mononuclear cell proliferation also when ADSCs were pre-exposed to the pro-inflammatory cytokine tumor necrosis factor alpha (TNFα). This reduction was more pronounced for the human cells. When cADSCs were co-cultured with allogenic PBMCs stimulated with LPS, a slight reduction on cPBMC proliferation was measured (Figure 9A). On the contrary, both naïve and pre-exposed hADSCs were able to significantly (*p* < 0.05) suppress proliferation of LPS-activated hPBMCs (Figure 9B).

**Figure 10:**
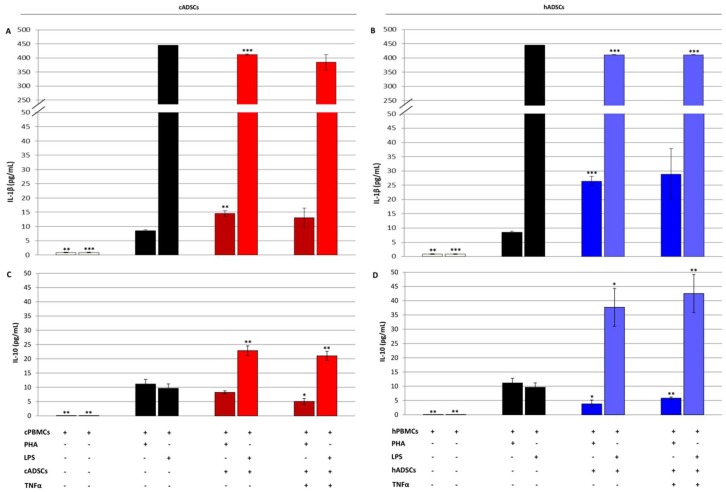
Cytokine production in the co-culture supernatant of canine and human ADSCs and allogenic PBMCs that were stimulated with PHA or LPS for three days. (**A**,**B**) Production of interleukin 1β (IL-1β) assessed in canine and human co-cultures, respectively, using sandwich ELISA kits. (**C**,**D**) Production of interleukin 10 (IL-10) measured in canine and human co-cultures, respectively, using sandwich ELISA kits. Data are expressed as mean ± SD (*n* = 3). * *p* < 0.05, ** *p* < 0.01, *** *p* < 0.001 indicate statistically significant difference compared to stimulated PBMCs.

To evaluate the effect of cADSCs and hADSCs on the activity of immune cells, production of interleukin 1β (IL-1β) and interleukin 10 (IL-10) in culture supernatant obtained from the co-cultures of ADSCs and PBMCs (1:10 ratio) was measured by commercial ELISA assays. The secretion of the pro-inflammatory cytokine IL-1β was higher both in the canine and human co-cultures when mononuclear cells were stimulated with PHA (Figure 10A,B). On the contrary, the level of IL-1β significantly decreased in the supernatant harvested from the co-culture of ADSCs and LPS-activated PMBCs (Figure 10A,B). Regarding IL-10, a decrease in its production was measured when cADSCs were co-cultured with PHA-activated cPBMCs, whereas an increase was detected in the case of PBMCs’ stimulation with LPS (Figure 10C). The cytokine profile for the human co-cultures showed a similar but emphasized trend compared to that of the canine counterpart (Figure 10D).

### 2.7. Cytological Evaluation

**Figure 11:**
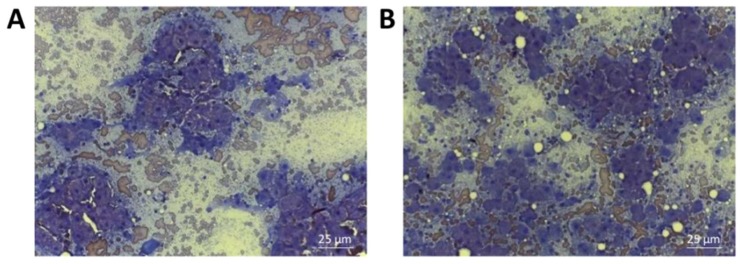
Representative images of cytological smears obtained from dogs affected by chronic liver disease. (**A**) Diff-Quik staining of hepatic smear performed before the first cell transplantation (magnification 40×). (**B**) Diff-Quik staining of hepatic smear performed 30 days after the second injection of cells (magnification 40×).

Cytological analysis was performed before the first cell transplantation and 30 days after the second injection. Both pre- and post-cell transplantation smears were of high cellularity and contained hepatocytes arranged in clusters of various size in a background of peripheral blood and protein. Most of the hepatocytes presented a round central or paracentral nucleus (Figure 11). In detail, cytological smears obtained before cell transplantation revealed hepatocytes characterized by a broad cytoplasm often of rarefied appearance towards cell periphery (Figure 11A). In addition, some spindle cells that sometimes infiltrated through hepatocytes and acted as a bridge between clusters were present. Inflammation was demonstrated by the presence of neutrophils, small and medium activated lymphocytes, and foamy macrophages. In cytological smears obtained 30 days after the second injection of cells, liver inflammation appeared diminished as well as the presence of spindle cells; moreover, the cytoplasm of hepatocytes appeared less rarefied (Figure 11B).

## 3. Discussion

Both in human and veterinary field, MSCs have generated great interest in regenerative medicine for their potential use in cell-transplantation and gene-therapy strategies also thanks to their anti-inflammatory and immunomodulatory properties. In the case of liver diseases, where the lack of donor organs, the risks associated with allotransplantations, and the necessary immunosuppression are objective limitations, the use of autologous MSCs for supporting liver regeneration appears a promising alternative. This is particularly true for ADSCs, since adipose tissue is sufficiently available in most people and tissue harvesting is a well-controlled and minimally invasive procedure [13]. In humans, liver diseases are clinically and pathologically highly comparable with their canine liver disease counterparts; consequently, dogs may represent a spontaneous and non-experimental animal model for human clinical investigations [14].

In this study, we compared several characteristics of canine and human MSCs isolated from adipose tissue samples, including morphology, surface antigen phenotype, proliferation, and in vitro differentiation potential. To the best of our knowledge, this is the first work that simultaneously compares specific morphological and functional characteristics of both canine and human MSCs derived from adipose tissue samples. Characterization of the two tissue sources was based on the minimal criteria formulated by the International Society for Cellular Therapy for defining multipotent MSCs [15]. These are plastic adherence under standard culture conditions, expression of surface markers such as CD79, CD90, and CD105 and lack of expression of CD14, CD34 and CD45, and multilineage differentiation potential in vitro [15].

Canine and human cell populations displayed similar in vitro spindle-shaped morphology, as evidenced by both phase-contrast images and staining of actin filaments with phalloidin. This fibroblast-like morphology was preserved through passaging in both cell populations. MSCs were further characterized according to their surface protein expression by flow cytometry. Human cells were found positive to the established MSCs markers CD73, CD90, and CD105. In addition to these, the population of human cells showed positivity to two other surface markers generally expressed by MSCs, CD29 and CD44 [16,17]. The canine cell population resulted positive to CD29, CD44, and CD90; as reported by others, expression of CD90 was lower than that of CD29 and CD44 [18,19]. Negative expression was instead observed for CD73 and CD105 surface markers. In this regard, it is important to consider that full characterization of MSCs from veterinary species is limited by the availability of species-specific antibodies [20]. Our results, however, are in agreement with those obtained by other groups, which have identified a panel of positive surface markers for characterizing canine MSCs by flow cytometry, which include CD29, CD44, and CD90 [18,21,22,23]. Flow cytometry immunophenotyping also demonstrated the negativity to CD14, CD34, and CD45, indicating the absence of hematopoietic cells in both stem cells populations.

The proliferation kinetics and growth rate of canine and human cells at different in vitro passages were further examined. The general trend was similar in the two cell populations, as proliferation rate decreased with increasing passage and culture time. Nevertheless, compared to hADSCs, cADSCs had significantly faster proliferation potential in vitro as reflected by the higher number of cell doublings in the population.

The proliferation rate is not the only criterion to consider for determining the abundance of MSCs in a cell population. The number and yield of MSCs that can be isolated from a single sample are also essential [24]. For this reason, we additionally measured the frequency of CFUs-F in cADSCs and hADSCs by a limiting dilution assay. This represents a useful method to establish the frequency of a well-defined cell in a population of cells [25]. We performed a CFUs-F assay to compare the CFUs potential between cADSCs and hADSCs after cell expansion at p1 and p3. This choice is meaningful in relation to the therapeutic use of stem cells, since early passages are considered appropriate for obtaining an adequate number of cells, safe in terms of chromosome alterations and genetic abnormalities, and, therefore, adequate for therapeutic clinical applications [26]. The CFUs-F frequencies for canine and human cells at p1 and p3 were similar; for both cell populations, frequency of colony formation slightly decreased at increasing passage, consistently with results reported in independent studies [27,28,29].

Osteogenic and adipogenic differentiation were then induced to evaluate multipotent differentiation potential of the ADSCs populations in vitro. When grown under appropriate conditions, both canine and human cells were able to differentiate into osteoblasts and adipocytes; nevertheless, some differences in differentiation potential were observed. In comparison to hADSCs, cADSCs accumulated more calcium deposits during osteogenic differentiation, as demonstrated by ARS staining. Histological data were supported by gene expression analysis, which confirmed a faster osteogenic differentiation in canine cells than their human counterparts. The osteogenic differentiation is initialized and precisely controlled temporally and spatially by RUNX2 [30]. It plays an essential role upstream in osteoblastic differentiation because it induces the expression of osteogenic extracellular matrix genes during osteoblast maturation, such as ALP, OPN, and OC [31]. After 21 days of differentiation, canine cells presented overexpression of these late markers and low levels of RUNX2 along with overexpression of OSX. OSX is a late-stage osteoblast-specific transcription factor which activates a repertoire of genes during differentiation of pre-osteoblasts into mature osteoblasts and osteocytes [32]. These findings suggest that after 21 days in ODM, cADSCs become mature osteoblasts. After the same culture time in ODM, human cells showed an overexpression of RUNX2, a low increase in OC, OPN, and OSX expression, and downregulation of ALPL. Considering mRNA expression of the osteoblast markers and the accumulation of calcium deposits, our data suggest a slower osteogenic differentiation potential of hADSCs than their canine counterpart.

The evaluation of the adipogenic commitment in canine and human cells was based on different differentiation protocols; nevertheless, the state-of-the-art protocol was specifically selected for each cell population. In addition to diverse media formulation, cADSCs underwent adipogenic induction for 21 days, compared to 14 days for hADSCs.

The conventional differentiation protocol for in vitro adipogenesis of human stem cells typically contains a mixture of insulin, isobutylmethylxanthine, indomethacin, and dexamethasone [33]. Initial work using the same protocol as commonly used for hADSCs resulted in limited adipogenesis of the canine cells (data not shown). Neupane and co-workers optimized the adipogenic differentiation medium for adipose-derived canine MSCs by replacing indomethacin with rosiglitazone, a member of the thiazolidinedione hypoglycemic drugs [34]. Recently, another group demonstrated that treatment of MSCs from different mammalian species to dexamethasone and rosiglitazone is necessary and sufficient for inducing their adipogenic differentiation [35]. Interestingly, these authors stated that dexamethasone and rosiglitazone drive adipogenic differentiation even in those species refractory to the conventional medium.

Accumulation of fat droplets in a cell’s cytoplasm is indicative of terminal adipogenic differentiation. In this sense, hADSCs appeared to have a superior adipogenic potential on the basis of a greater number of positively stained lipid vacuoles, compared with the canine MSCs. These results are consistent with those described by others, where few and small lipid vacuoles were observable following adipose differentiation of canine MSCs [36,37]. Nevertheless, classical histochemical staining for detecting adipose differentiation has to be considered in parallel to the measurement of genic expression markers. When comparing gene expression profiles, PPARG and CEBPA mRNAs were similarly upregulated following adipogenic induction in both canine and human cells. These are two key adipogenic transcription factors responsible for inducing and maintaining the adipose phenotype [38]. Also, the adipocyte marker FABP4 showed a comparable overexpression in the two cell populations subjected to adipogenic stimuli. On the contrary, hADSCs showed the higher shift in the expression of ADIPOQ, an adipokine which is a marker of final maturation, therefore indicative of functional adipocyte [35]. The same consideration can be done for GLUT4, the insulin-regulated glucose transporter whose expression is exclusive of adipocytes which have completed their maturation and which would be in the final stages of adipogenesis [39].

One of the main challenges for using stem cells in clinical applications is to ensure their survival and functionality during delivery from the laboratory to the administration site [40]. Although cryopreservation is the best choice for long-term storage of MSCs, these cells may be maintained in a non-frozen state for short-term shipment. Different studies agree with the fact that PBS is a good medium for suspending cells to be transplanted thanks to its stronger pH buffering capacity over other solutions, such as saline or dextrose [41,42]. Among other parameters, time and temperature mainly affect MSCs number during shipping conditions [43]. In this work, we found that a high viability (>80%) of cADSCs suspended in PBS and maintained at a controlled temperature can be assured up to 72 h; hADSCs viability exposed to the same conditions decreased over time more quickly.

MSCs represent an exceptional resource not only for their potential ability to induce tissue regeneration, but also for their immunoregulatory properties. In this study, the paracrine effects of stem cells were investigated after the transfusion of autologous ADSCs in dogs with spontaneous liver disease. Currently, several experimental animal models of liver injury exist and are described in the literature [44]. In these studies, the most commonly used approach to induce experimental liver injury in mice or rats is the periodic administration of toxic agents, such as carbon tetrachloride (CCl4) or thioacetamide (TAA) [8,45,46]. Although the CCl4 and TAA treatments generate well-established animal models of hepatic disease that show pathological characteristics consistent with that of acute liver failure, these have a number of disadvantages that limit the conclusions that can be drawn from these studies [44]. Therefore, all experimental findings need to be critically evaluated in appropriate models that reflect the pathological mechanisms of human hepatic disorders before their translation into clinical treatments.

Compared to laboratory animals, anatomy and physiology in dogs, as well as the onset of spontaneous diseases with similar pathogenesis, more closely resemble humans [47]. Additionally, dogs live longer in environments similar to humans, and are therefore exposed to similar external factors underlying common disease states. As a consequence, naturally occurring diseases in veterinary species represent suitable models of human disease because these reflect the complex physiologic, genetic, and environmental variation found in the human population [48].

In dogs, allogeneic ADSCs have recently and successfully been used to treat liver injury during the acute phase [49]. However, healthy dogs were involved in that study, and acute hepatic injury was experimentally induced by injection of CCl4. Our work is the first to use autologous ADSCs in dogs bearing natural occurring chronic liver damage. We believe that this study could have a huge clinical significance not only for evaluating the efficacy of stem cell therapies ultimately intended to humans, but also because chronically liver injured animals are thought to be the major population to benefit of a future cell therapy-based treatment for hepatic disease. Indeed, as mentioned above, chronic hepatitis in dogs presents with non-specific clinical signs, and the diagnosis is confirmed by histological examination only at advanced stages [12].

In the work presented here, dogs with spontaneous chronic hepatic disease were used and transplanted with undifferentiated autologous cADSCs through portal vein under ultrasound guidance. Previous studies have shown that portal and splenic injections of MSCs are the preferred administration routes when hepatic injury must be treated, since both of them result in homogeneous distribution of stem cells in the liver with a high hepatic uptake [49,50]. On the contrary, systemic intravenous injection leads to almost all cells trapped in the lungs, although MSCs then possess homing behavior for the recruitment in the wound site.

Serum levels of AST, ALT, and ALP enzymes are typically measured clinically as biomarkers for liver injury; serum albumin is instead a marker of liver function [51]. Recently, other serum and plasma biomarkers have been reported as potential indicators of liver injury [51]. For example, elevated serum levels of BAs were measured in different forms of liver injury in rodents [52,53]. In our study, ALT, AST, ALP, and total BAs levels were found markedly high before treatments with autologous cADSCs, whereas these showed a significant reduction after cell transplantations. The levels of the markers examined in this study remained high in dogs that received PBS instead of cells. These finding are in agreement with previous studies using rodents, in which reduction in serum levels of ALT and AST was recorded after MSCs injection [54,55,56]. Most importantly, we did not observe negative side effects of cell transplantation, consistently with the results of others [9,57]. Considering the decrease in serum biochemical parameters after injection of autologous ADSCs in dogs, we could hypothesize that soluble factors secreted from these cells might mainly contribute to recovery of hepatic injury and improvement in liver function.

In effect, over the past decade it has become clear that MSCs interact with cells of the immune system and modulate their function by both direct cell-to-cell contact and through the secretion of different mediators [49,58]. The secretion of such a broad range of bioactive molecules is now believed to be the main mechanism by which MSCs achieve their therapeutic effect in different pathological conditions [58]. If, however, many of the details underlying the mechanisms by which MSCs modulate the immune response have been defined for human and rodent MSCs, only a few works on canine ADSCs immunomodulation have been published so far, and these report contradictory conclusions [19,21,23,59]. Although it was not the purpose of the current study, we believe that further study is needed to identify the detailed mechanisms responsible for the immunomodulatory effects of cADSCs we observed in vivo.

With the aim of establishing whether the beneficial effect we observed in vivo could be attributed to the immunomodulatory properties of stem cells, canine and human ADSCs were co-cultured with PBMCs isolated from allogenic unrelated donors at a 1:10 ratio. Previous studies have demonstrated that inhibition of immune cell proliferation by MSCs has no immunological restriction; similar suppressive effects have been observed towards autologous or allogenic responder cells [60,61]. The immunomodulatory properties of MSCs have been widely described based in large part on assays that evaluate suppression of T cell proliferation [23]. However, over the years, it has been evidenced that MSCs affect not only T cells but also other cells of the immune system [62,63]. For this reason, two different molecules were used in this study to stimulate PBMCs, PHA and LPS. PHA is a mitogen known to activate T cell proliferation in several mammalian species, whereas LPS is a bacterial endotoxin mainly responsible for the activation of B-cells, monocytes, vascular cells, and polymorphonuclear cells [64,65]. Several works also suggest that the immunomodulatory effects of MSCs are determined by the local conditions of the microenvironment [58,66]. Therefore, whether the in vivo use of MSCs for therapeutic applications does not require priming of these cells; in vitro, MSCs need to be pre-exposed to mediators present in inflammatory environments—such as interferon gamma (IFNγ), TNFα, or IL-1β—in order to efficiently modulate immune cell function [66].

In agreement with another work, we found suppressive effects of cADSCs on allogenic PBMCs proliferation following induction with the mitogen PHA after three days of co-culture [67]. This reduction was not significant when mononuclear cells were treated with LPS. Pre-treatment of cADSCs with TNFα further enhanced the ability of stem cells to decrease allogenic PBMCs proliferation after stimulation with both PHA and LPS. With regard to human cells, a significant suppression of PBMCs proliferation by allogenic stem cells was observed for both the stimulators used, and higher inhibition resulted after hADSCs were pre-exposed to TNFα. The evidence that pre-treatment of MSCs with pro-inflammatory cytokines enhances their suppressive effect on PBMCs is in agreement with previous studies performed in rodents [66,68]. Based on our data, we may assume that ADSCs successfully inhibit T cell proliferation, but exert only a moderate effect on the proliferation of other immune cell populations in the dog.

As reported above, the immunomodulatory effects of MSCs are jointly executed by both direct cell-to-cell contact and soluble factors secretion [49,58]. In this study, we evaluated the secretion profile of the pro-inflammatory cytokine IL-1β, and of IL-10, one of the most important immunoregulatory and anti-inflammatory cytokines [69]. Canine PBMCs’ stimulation with PHA determined an increase in the production of both IL-1β and IL-10; at baseline, neither unstimulated PBMCs nor ADSCs produced these mediators (data not shown). The presence of cADSCs resulted in an increase of IL-1β level, which was significant only when stem cells were not pre-exposed to TNFα. Unexpectedly, a slight reduction of IL-10 emerged in the co-culture of cADSCs with PHA-stimulated cPBMCs. A similar scenario was observed with the human cells. In this regard, it is necessary to point out that contradictory results are reported in the literature. For example, in agreement with our results, in the study of Bertolo and co-workers, the presence of human MSCs inhibited the production of IL-10 [70]. Conversely, IL-10 expression was significantly boosted in the human MSCs-PBMCs co-culture after PHA-stimulation [71]. Still, in the work of Rasmusson and colleagues, IL-10 levels did not change when human MSCs were added to allogenic PBMCs stimulated with PHA [72]. In another study, IL-10 production was not different when canine immune cells where cultured with or without cADSCs [59]. In that work, however, activators other than PHA were used for PBMCs stimulation. These controversial results could be explained by the fact that different activators were used in the various studies, at different concentrations, in addition to having diverse MSCs/PBMCs ratios.

When evaluating the secretion profile of these interleukins in PBMCs after stimulation with LPS, high levels of both IL-1β and IL-10 were measured, in agreement with previous works [73,74]. As before, unstimulated PBMCs and ADSCs did not secrete these cytokines when cultured alone (data not shown). Interestingly, co-culture of LPS-stimulated PBMCs with allogenic ADSCs determined a significant decrease of IL-1β, and a concomitant increase in IL-10 level both for canine and human cell populations. The production of IL-10 is believed to be related to an increased release of prostaglandin E2 (PGE2) from MSCs, which acts on macrophages receptors stimulating the secretion of this anti-inflammatory cytokine [75]. It is interesting to note that PGE2 synthesis seems to be promoted by IL-1β released by monocytes and macrophages activated by LPS [76,77]. These results would indicate that ADSCs, in response to high levels of IL-1β, favor the polarization of macrophages into the IL-10-producing anti-inflammatory phenotype.

Taken together, our data would suggest that ADSCs possess an immunomodulatory effect, although different mechanisms are used towards the various components of the immune system. cADSCs appear to be involved in suppressing T cell proliferation, as emerged when allogenic PBMCs were stimulated with PHA, whereas these mainly act on modulating the cytokines secretion profile of other immune cells, as seen in LPS-activated PBMCs. Consequently, the immunomodulatory effect of MSCs seems to be mediated by a network of molecular pathways rather than being dependent on a single mechanism. We believe that our in vitro results provided a valuable indication of the immunomodulatory effect of ADSCs; however, further studies are needed to identify the detailed mechanisms. Since similar results between dogs and humans were obtained, we believe that this work can have clinical relevance and a translational potential.

Nonetheless, in order to determine whether cADSCs transplantation generated beneficial and durable effects for the dogs, we analyzed cytological smears obtained from diseased liver before the first cell transplantation and 30 days after the second injection. Cytological evaluation is a minimally invasive analysis used to investigate the hepatic lesions, often fundamental to formulate a diagnosis together with the histopathological examination [78]. Cytological smears obtained before cell injection showed inflammatory cells along with a substantial amount of hepatocytes showing rarefied cytoplasm towards cell periphery; these were accompanied by some spindle cells through hepatocyte clusters. Such a description is compatible with liver fibrosis, which was indeed diagnosed [79]. After 30 days from the second cell treatment, cytological analysis was repeated. A decrease in inflammatory cells as well as in spindle cells was revealed in the smears. Furthermore, hepatocyte cytoplasm appeared less rarefied compared to that of cytological specimens before cell treatment. A reduction in the inflammatory response seems to agree with the fact that MSCs immunomodulation could be one of the mechanisms responsible for the improvement of liver disease.

## 4. Materials and Methods

### 4.1. Isolation and Culture of Canine and Human ADSCs

hADSCs were isolated from human abdominal fat of healthy patients (age: 21–36; BMI: 30–38) undergoing cosmetic surgery procedures, following the guidelines of the University of Padova’s Plastic Surgery Clinic. cADSCs were isolated from fat specimens (10 cc) collected from the suprascapular or interscapular region with aseptic techniques and regional anesthesia. The Ethical Committee of Padova Hospital approved the research protocol (5 March 2009; 20150). Written informed consent was obtained from all patients, in accordance with the Helsinki Declaration. All the animals were treated and handled in accordance with the guidelines on minimum health requirements for the use of stem cells in veterinary medicine approved by the Italian Ministry of Health (17 October 2013, 277).

Human adipose tissue was processed as previously described [80]; canine biopsies followed the same procedure. Briefly, tissues were washed with PBS (EuroClone, Milan, Italy), minced, and digested with 0.075% collagenase type II from *Clostridium histolyticum* (Sigma-Aldrich, Saint Louis, MO, USA) in Hanks’ balanced salts solution (HBSS; EuroClone) for 3 h at room temperature (RT) with shaking. Cells from the stromal vascular fraction were pelleted and rinsed with PBS. Red blood cells contamination was deleted by a step in red blood cells lysis buffer (Sigma-Aldrich) run for 10 min at RT. The resulting viable cells were maintained at 37 °C and 5% CO2 in BM, consisting of Dulbecco’s modified Eagle’s medium (DMEM) high glucose (EuroClone) supplemented with 10% fetal bovine serum (FBS; EuroClone), and 1% penicillin/streptomycin (P/S; EuroClone). Culture medium was refreshed twice a week. At 80–90% confluence, both cell populations were detached with trypsin-EDTA solution (Sigma-Aldrich) and passaged repeatedly.

### 4.2. Immunofluorescence Analysis

For immunofluorescence staining, 2 × 10^4^ human and canine cells at p1, p3, and p5 were seeded on glass coverslips put into 24-well plates and cultured in BM. Twenty-four h after seeding, cells were fixed in 4% paraformaldehyde solution in PBS for 10 min, then permeabilized for 10 min in 0.5% Triton X-100 (Sigma-Aldrich) prepared in PBS. After three washing with PBS, the cells were incubated in 2% bovine serum albumin (BSA; Sigma-Aldrich) solution in PBS for 1 h at RT. The cells were then stained with 5 µg/mL phalloidin tetramethylrhodamine B isothiocyanate (Sigma-Aldrich) for 40 min at RT. Nuclear staining was performed with 2 μg/mL Hoechst H33342 (Sigma-Aldrich) solution for 15 min. The cells were observed with the LeicaDM5000 B microscope (Imaging Facility, Department of Biology, University of Padova, Padova, Italy).

### 4.3. Flow Cytometry

Adherent cells from p3 were dissociated and resuspended in flow cytometry staining buffer (R&D Systems, Minneapolis, MN, USA) at a final cell concentration of 1 × 10^6^ cells/mL. Human cells were incubated with the following fluorescent monoclonal mouse anti-human antibodies: CD29 APC (Thermo Fisher Scientific, San Diego, CA, USA); CD44 FITC (Thermo Fisher Scientific); CD73 APC (eBioscience^TM^, Thermo Fisher Scientific); CD90 BV510 (BD Biosciences, San Jose, CA, USA); CD105 PE-Cyanine7 (eBioscienceTM); CD14 PE (eBioscienceTM); CD34 APC-eFluor 780 (eBioscienceTM); CD45 Pacific Orange (eBioscienceTM). Canine cells were incubated with the following fluorescent monoclonal mouse antibodies: CD29 APC (Thermo Fisher Scientific); CD44 FITC (Thermo Fisher Scientific); CD73 APC (eBioscienceTM); CD90 APC (eBioscienceTM); CD105 PE-Cyanine7 (eBioscienceTM); CD14 PE (Thermo Fisher Scientific); CD34 PE (eBioscienceTM); CD45 FITC (eBioscienceTM). Cells were washed twice with 2 mL of flow cytometry staining buffer and resuspended in 500 μL of flow cytometry staining buffer. Fluorescence was evaluated by flow cytometry in Attune NxT flow cytometer (Thermo Fisher Scientific). Data were analyzed using Attune NxT software (Thermo Fisher Scientific).

### 4.4. MTT Assay

Cell proliferation rate was investigated through the MTT-based assay according to the method previously described [81]. Briefly, 2 × 10^4^ cells at p1, p3, and p5 were seeded onto 24-well plates in BM. After one, three, and seven days from seeding, culture medium was harvested and cells were incubated for 3 h at 37 °C in 1 mL of 0.5 mg/mL MTT solution prepared in PBS. After removal of the MTT solution, 0.5 mL of 10% dimethyl sulfoxide in isopropanol was added for 15 min at RT with gently rotation. For each sample, optical density (OD) values at 570 nm were recorded in duplicate using a multilabel plate reader (Victor 3, Perkin Elmer, Milan, Italy).

### 4.5. PD Assay

In order to establish growth potential of cADSCs and hADSCs, 1.2 × 10^5^ cells at p2 were seeded into 6-well plates. Every three days, cells were detached, counted, and seeded again at the same density in a new 6-well plate. This was repeated until the cells reached p6. The PD of the cells was calculated according to the formula [82]

PD = (logNt − logN0)/log2(1)

where PD represents the number of cell divisions that occur in each passage; Nt corresponds to cell number on the third day, and N0 is the initial seeding number of cells. To determine the cumulative PD, the PD level for each passage was calculated, then added to the levels of the previous passages.

### 4.6. Limiting Dilution CFUs-F Assay

In order to determine MSCs frequency in the canine and human cell populations, a limiting dilution CFUs-F assay was performed, as published elsewhere [27]. Briefly, 5 × 10^3^, 2.5 × 10^3^, 1.25 × 10^3^, 6.25 × 10^2^, 3.12 × 10^2^, or 1.56 × 10^2^ cADSCs and hADSCs at p1 and p3 were each seeded in six wells in a 96-well plate, with each row containing wells of the same cell density. Cells were cultured in BM for eight days. CFUs-F colonies were then fixed in 4% paraformaldehyde solution, and stained with 0.1% toluidine blue prepared in 1% paraformaldehyde solution for 20 min at RT. The number of negative and positive wells was determined for each cell concentration, considering a positive well as one containing ≥20 toluidine blue-stained cells. The percentage of wells negative for CFUs was used to calculate the frequency of CFUs-F according to the formula [83]

F = e^−*x*^ (2)

where F is the ratio of negative to positive wells within a row, e is the natural logarithm constant 2.71, and *x* is the number of CFUs per well. Based on a Poisson distribution of a clonal-cell lineage, the value F = 0.37 occurs when the number of total canine or human ADSCs plated in a well includes a single CFU.

### 4.7. In Vitro Differentiation of cADSCs and hADSCs

For osteogenic differentiation, 2 × 10^4^ canine or human cells at p3 were seeded onto 24-well plates and incubated in ODM for 21 days. ODM was made of DMEM High Glucose supplemented with 10% FBS, 1% P/S, 10 ng/mL Fibroblast Growth Factor 2 (ProSpec, East Brunswick, NJ, USA), 10 mM β-glycerophosphate (Sigma-Aldrich), and 10 nM dexamethasone (Sigma-Aldrich). The medium was replenished twice a week.

For adipogenic differentiation of canine cells, 2 × 10^4^ cADSCs at p3 were seeded onto 24-well plates and incubated in canine cADM up to 21 days. cADM was made of DMEM high glucose supplemented with 10% FBS, 1% P/S, 10 µM rosiglitazone (Sigma-Aldrich), and 1 µM dexamethasone (Sigma-Aldrich). For adipogenic differentiation of human cells, 2 × 10^4^ hADSCs at p3 were seeded onto 24-well plates and cultured in hADM for 14 days. hADM consisted of DMEM high glucose supplemented with 10% FBS, 1% P/S, 10 µg/mL insulin (Sigma-Aldrich), 0.5 mM isobutylmethylxanthine (Sigma-Aldrich), 0.1 mM indomethacin (Sigma-Aldrich), and 1 µM dexamethasone (Sigma-Aldrich). Both differentiation media were changed twice a week.

### 4.8. ARS Staining and Quantification

Mineral calcium deposits were visualized and quantified by means of ARS staining after 21 days from cell seeding, as published elsewhere [84]. In detail, cells were fixed in 4% paraformaldehyde solution in PBS for 10 min at RT, then washed twice with PBS. For ARS staining, cells were incubated with 0.5 mL of 40 mM ARS (Sigma-Aldrich) solution pH 4.2 for 20 min at RT with gentle shaking. After four washes with distilled water, phase-contrast images were taken. Then, cells were washed with PBS and incubated with 0.5 mL of 10% (*w*/*v*) Cetylpyridinium Chloride (CPC; Sigma-Aldrich) in 10 mM sodium phosphate solution pH 7.0 for 20 min at RT with gentle agitation. For each sample, OD values at 570 nm were measured with Victor 3 plate reader.

### 4.9. ORO Staining and Quantification

The intracellular lipid content was assessed by means of ORO staining and quantification after 14 and 21 days of adipogenic differentiation for human and canine cells, respectively, as published elsewhere [85]. Briefly, cells were fixed in 4% paraformaldehyde solution in PBS for 10 min at RT, then washed twice with PBS. Then, ORO stock solution was prepared by dissolving 3.5 mg/mL of ORO powder (Sigma-Aldrich) in isopropanol. ORO working solution was then made by adding three parts of ORO stock solution to two parts of distilled water. Cells were stained with 0.5 mL of fresh ORO working solution for 15 min at RT. After four washes with distilled water, phase-contrast images were taken and ORO staining was extracted with 0.25 mL 100% isopropanol. For each sample, OD values at 490 nm were recorded with Victor 3 multilabel plate reader.

### 4.10. Real-Time PCR

Total RNA was isolated from human and canine cells after 14 days or 21 days of differentiation with Total RNA Purification Plus Kit (Norgen Biotek Corporation, Thorold, Canada). The RNA samples quality and concentration were measured using the NanoDrop™ ND-1000 (Thermo Fisher Scientific). The cDNA was synthesized starting from 500 ng of total RNA of each sample with the SensiFASTTM cDNA Synthesis kit (Bioline GmbH, Germany) in a LifePro Thermal Cycler (Bioer Technology, China). Real-time PCR of genes involved in osteogenic and adipogenic differentiation was performed. Canine (Table 3) and human (Table 4) primers were selected for each target gene with Primer 3 software. Real-time PCR was carried out using the designed primers at a concentration of 400 nM and SensiFASTTM SYBR No-ROX mix (Bioline GmbH) on a Rotor-Gene 3000 (Corbett Research, Sydney, Australia). Thermal cycling conditions were as follows: denaturation at 95 °C for 2 min, followed by 40 cycles of denaturation at 95 °C for 5 s; annealing at 60 °C for 10 s; and elongation at 72 °C for 20 s. Data analysis was performed using the ΔΔ*C*_t_ method [86] using transferrin receptor (TFRC) as internal reference. Results were reported as fold regulation of target genes in test group (cells grown in differentiation medium) compared with control group (cells cultured in BM).

### 4.11. Cell Preservation under Transportation Conditions

In order to test cell viability under transportation conditions, 2 × 10^5^ and 4 × 10^5^ cADSCs or hADSCs at p3 were resuspended in PBS in 2 mL cryogenic vials, then stored in a polystyrene box containing dry ice up to 72 h at RT. Every 24 h, cells were counted using a Countess II automated cell counter (Thermo Fisher Scientific). After the mimicked three days’ shipment, cells from the cryovials were recovered, seeded into 96-well plates, and imaged under a phase-contrast microscope after additional one and three days of culture in BM.

### 4.12. Transplantation of Autologous cADSCs

Ten dogs with severe degenerative hepatopathy were included in this study. Dogs with cancer in liver or other organs were excluded. Thrombosis of the hepatic vein was another criteria of exclusion. For the transplantation, autologous cells (5 × 10^5^ cells/kg) suspended in 2 mL PBS were transfused through the portal vein under ultrasound guidance, after sedation with medetomidine (20 μg/kg by intravenous injection). Low passage (p2–p5) cADSCs were used for injection. After 30 days from the inoculum, each dog underwent a second transplantation of autologous cells. The control group was injected with the same volume of PBS without cells.

Blood samples were obtained from the jugular vein for measuring levels of liver biomarkers before transplantation and seven days after each of the two cells injections. In particular, levels of serum ALT/GPT, AST/GOT, ALP, albumin, and total BAs were measured using an automated clinical chemistry analyzer (Fuji Dri-Chem, Fujifilm, Japan).

Hepatic cytological specimens were obtained by ultrasound guided fine-needle capillary sampling without aspiration before the first cell injection and 30 days after the second treatment. Smears were air-dried, stained using the Diff-Quick staining and then imaged under a light microscope.

### 4.13. PBMCs Proliferation Assay and Cytokine Production Analysis

Canine and human PBMCs were isolated from fresh whole blood collected from healthy dogs and healthy volunteers, respectively, through density gradient separation with 1.077 g/mL Ficoll-Paque™ PLUS (GE Healthcare Life Sciences, Little Chalfont, UK) according to the manufacturer’s protocol. The mononuclear cells located at the interface between Ficoll and plasma-medium layer were washed twice with PBS, suspended in RPMI 1640 medium (EuroClone) containing 10% FBS and 1% P/S, then counted using the Countess II Automated Cell Counter.

For the proliferation assay, cADSCs and hADSCs were used as stimulators, while allogenic canine and human PBMCs were used as responders. In detail, 2.5 × 10^3^ cADSCs or hADSCs at p3 were plated in triplicates in 200 μL RPMI 1640 medium onto 96-well plates. One day after seeding, the cells were inactivated by treatment with 15 μg/mL mitomycin C (Sigma-Aldrich) for 2 h at 37 °C. Then, 2.5 × 10^4^ allogenic cPBMCs or hPBMCs were co-cultured with the corresponding stem cell populations (10:1 ratio) under mitogenic stimulation with 5 μg/mL PHA (Sigma-Aldrich) or 100 ng/mL LPS (from *Escherichia coli* 0111:B4; Sigma-Aldrich).

After three days, 50 μL of the supernatant from each well of the co-cultures were collected, centrifuged at 1400 rpm for 1 min, then stored frozen for analysis of IL-1β and IL-10 production using commercial sandwich ELISA kits (Thermo Fisher Scientific) according to the manufacturer’s instructions. The proliferation of canine and human PBMCs was analyzed using an MTS Cell Proliferation Assay Kit (Colorimetric) (Abcam, Cambridge, UK) according to the manufacturer’s protocol. OD values at 490 nm were measured using Victor 3 plate reader 3 h after incubation. PBMCs proliferation was calculated as proliferation percentage normalized to the value measured for stimulated PBMCs, which was assigned a value of 100% [23].

### 4.14. Statistical Analyses

Each cell type was plated in triplicates with appropriate controls. Each experiment was performed independently three times. Results were expressed as mean ± standard deviation (SD). Effects of differentiation media were presented as fold-change relative to control (cells in BM). Data from the animals (*n* = 10) were pooled for statistical analysis performed with GraphPad Prism v6 (GraphPad Software Inc., La Jolla, CA). Comparative analysis was performed by two-way analysis of variance followed by post-hoc Bonferroni test. Student’s *t*-test was performed to determine the statistical significance between two groups. Statistical significance was set at *p* < 0.05; different labels indicate * *p*  <  0.05, ** *p*  <  0.01, and *** *p*  <  0.001.

## 5. Conclusions

The transplantation of undifferentiated autologous ADSCs could represent a possible future therapy for treating liver diseases, both in human and veterinary field. In this study, several characteristics of cADSCs have been investigated and compared to that of their human counterparts. Few differences are evidenced between the two stem cell populations with regard to morphology, immunophenotyping, and proliferation. Major differences are found in the in vitro differentiation potential, with the cADSCs showing greater osteogenic potential and lower adipogenic potential than human cells. For cell-therapy purposes, it is fundamental to ensure the viability and functionality of stem cells during short-term delivery. Our results show that controlled temperature constitutes an appropriate condition for maintaining the viability of MSCs up to 72 h, particularly cADSCs, and that PBS represents a proper cell suspension medium. When examining the efficacy of autologous cADSCs transplantation in dogs with severe liver diseases, a significant reduction in biomarkers levels of liver injury—such as AST, ALT, ALP, and total BAs—is observed, indicating an improvement of liver function. This beneficial effect could be attributed to the immunomodulatory properties of stem cells, as evidenced by their ability to inhibit T cell proliferation and modulate cytokines secretion by other immune cells. Evaluation of cytological specimens after cADSCs injections seems to confirm the beneficial effect of the stem cell-based therapy, as revealed by a decrease in inflammatory and spindle cells, as well as by the appearance of hepatocytes. Future clinical studies and comprehension of the molecular mechanisms are, however, required to advance understanding of the therapeutic effects of cADSCs in the treatment of liver diseases.

Overall, we believe that the correlations between canine and human ADSCs described in this study might be helpful not only for the development of veterinary clinical applications but also for the opportunity to translate the results to the human clinical practice in future.

## Figures and Tables

**Table 1:** Surface marker expression of cADSCs and hADSCs

Surface Marker	cADSCs	hADSCs
CD29	99.714 ± 0.130	95.481 ± 0.325
CD44	99.206 ± 0.105	99.811 ± 0.075
CD73	0.011 ± 0.005	98.938 ± 0.025
CD90	93.480 ± 0.350	99.299 ± 0.020
CD105	0.013 ± 0.010	99.649 ± 0.025
CD14	0.163 ± 0.000	0.045 ± 0.000
CD34	0.006 ± 0.100	0.388 ± 0.250
CD45	0.000 ± 0.000	0.000 ± 0.000

Data are displayed as percentages expressed as mean ± standard deviation (SD).

**Table 2:** Frequency of CFUs-F (mean ± SD) for cADSCs and hADSCs at different passages

CFUs-F	cADSCs	hADSCs
p1	1/(1.92 × 10^3^ ± 27)	1/(1.86 × 10^3^ ± 32)
p3	1/(2.34 × 10^3^ ± 26)	1/(2.18 × 10^3^ ± 28)

**Table 3:** Canine primer sequences.

Gene	Sequence FOR (5′–3′)	Sequence REV (5′–3′)	Length (bp)
*cADIPOQ*	CCGTTCGGCATTCAGTGTG	TGTCTTTCTTGTAGAGGCTGACC	206
*cALPL*	CATTGCCCACTCCTCTCTGAA	GTTTCTTTCTCTTCCTCACTGACCA	167
*cCEBPA*	GCCTTGGAAACGCAAACTCG	GTCCCTGTATGTCCTCCCTTC	219
*cFABP4*	CCATCCTATTCTAGACCGTTGAGAG	GCCACCATAAGAACATTTGCATCA	211
*cGLUT4*	TCTCCTGCTCGCCTTCTTC	CCTAAGTAATCGAGTTCCGTGCTG	147
*cOC*	CCTTTGGGATTTGGCGTCC	GGTTCTGTCTGGGTCTGTGAG	199
*cOPN*	TGACTTAGATGACGACTCCAACG	TGGGACTTCTTAGATTTGGACCTC	174
*cOSX*	ACGACACTGGGCAAAGCAG	ATGTCCAGGGAGGTGTAGAC	285
*cPPARG*	GCAGGAGATCACAGAGTACGC	CATCAAGGACGCCAGCATC	124
*cRANKL*	ACACTGATGAAAGGAGGTAGCAC	TGTTGCATCTTGATCTGGGTCC	159
*cRUNX2*	GCAAGTTCCAGCAGATCGC	GTGGTTGTCAGGAGTGGTCA	168
*cTFRC*	AGCCACCTCCAGACTAACG	GCAGAGTGTGAGAGCCAGA	176

ADIPOQ, adiponectin; ALPL, alkaline phosphatase, liver/bone/kidney; CEBPA, CCAAT enhancer binding protein alpha; FABP4, fatty acid binding protein 4; GLUT4, solute carrier family 2 (facilitated glucose transporter), member 4; OC, osteocalcin; OPN, osteopontin; OSX, osterix; PPARG, peroxisome proliferator activated receptor gamma; RANKL, TNF superfamily member 11; RUNX2, runt related transcription factor 2; TFRC, transferrin receptor.

**Table 4:** Human primer sequences.

Gene	Sequence FOR (5′–3′)	Sequence REV (5′–3′)	Length (bp)
*hADIPOQ*	GTTGTGTGCCTGTTTCTGACC	GCATCTATCATCCACTCTCCTATTTCTG	153
*hALPL*	GGCTTCTTCTTGCTGGTGGA	CAAATGTGAAGACGTGGGAATGG	181
*hCEBPA*	GGACTTGGTGCGTCTAAGATGAG	GCATTGGAGCGGTGAGTTTG	147
*hFABP4*	TGACCTGGACTGAAGTTCGC	AAGCACAATGAATACATCATTACATCACC	193
*hGLUT4*	CCAGTATGTTGCGGAGGCTA	TCAAGTTCTGTGCTGGGTTTCA	189
*hOC*	GCAGCGAGGTAGTGAAGAGAC	AGCAGAGCGACACCCTA	193
*hOPN*	TGGAAAGCGAGGAGTTGAATGG	GCTCATTGCTCTCATCATTGGC	192
*hOSX*	TCAGAATCTCAGTTGATAGGGTTTCTC	GGGTACATTCCAGTCCTTCTCC	183
*hPPARG*	CAGGAGATCACAGAGTATGCCAA	TCCCTTGTCATGAAGCCTTGG	173
*hRANKL*	TCAGCATCGAGGTCTCCAAC	CCATGCCTCTTAGTAGTCTCACA	194
*hRUNX2*	AGCCTTACCAAACAACACAACAG	CCATATGTCCTCTCAGCTCAGC	175
*hTFRC*	TGTTTGTCATAGGGCAGTTGGAA	ACACCCGAACCAGGAATCTC	222

## Abbreviations

MSCs	mesenchymal stem cells
hADSCs	human adipose-derived stem cells
cADSCs	canine adipose-derived stem cells
BM	basal medium
ODM	osteogenic differentiation medium
hADM	human adipogenic differentiation medium
cADM	canine adipogenic differentiation medium
